# Early Prediction of Therapy Response to Abiraterone Acetate Using PSA Subforms in Patients with Castration Resistant Prostate Cancer

**DOI:** 10.3390/ijms17091520

**Published:** 2016-09-09

**Authors:** Katrin Schlack, Laura-Maria Krabbe, Manfred Fobker, Andres Jan Schrader, Axel Semjonow, Martin Boegemann

**Affiliations:** 1 Department of Urology, Prostate Center, University Hospital Muenster, Albert-Schweitzer-Campus 1, GB A1, Muenster D-48149, Germany; laura-maria.krabbe@ukmuenster.de (L.-M.K.); andresjan.schrader@ukmuenster.de (A.J.S.); axel.semjonow@ukmuenster.de (A.S.); martin.boegemann@ukmuenster.de (M.B.); 2 Department of Urology, University of Texas Southwestern Medical Center, Dallas, TX 75390-9110, USA; 3 Center for Laboratory Medicine, University Hospital Muenster, Albert-Schweitzer-Campus 1, GB A1, Muenster D-48149, Germany; manfred.fobker@ukmuenster.de

**Keywords:** mCRPC, surrogate biomarker, abiraterone acetate, prognosticators, prostate cancer, [−2]proPSA, fPSA, PHI, tPSA

## Abstract

The purpose of this study was to evaluate the prognostic ability of early changes of total prostate specific antigen (tPSA), free PSA (fPSA), [−2]proPSA and the Prostate Health Index (PHI) following initiation of Abiraterone-therapy in men with castration resistant prostate cancer (mCRPC). In 25 patients, PSA-subforms were analyzed before and at 8–12 weeks under therapy as prognosticators of progression-free-survival (PFS) and overall survival (OS). Comparing patients with a PFS < vs. ≥12 months by using Mann–Whitney–Wilcoxon Tests, the relative-median-change of tPSA (−0.1% vs. −86.8%; *p* = 0.02), fPSA (12.1% vs. −55.3%; *p* = 0.03) and [−2]proPSA (8.1% vs. −59.3%; *p* = 0.05) differed significantly. For men with ≤ vs. >15 months of OS there was a non-significant trend for a difference in the relative-median-change of fPSA (17.0% vs. −46.3%; *p* = 0.06). In Kaplan–Meier analyses, declining fPSA and [−2]proPSA were associated with a longer median PFS (13 months, 95% confidence interval (CI): 9.6–16.4 vs. 10 months, 95% CI: 3.5–16.5; *p* = 0.11), respectively. Correspondingly, decreasing fPSA and [−2]proPSA values indicated an OS of 32 months (95% CI: not reached (NR)) compared to 21 months in men with rising values (95% CI: 7.7–34.3; *p* = 0.14), respectively. We concluded that the addition of fPSA- and [−2]proPSA-changes to tPSA-information might be further studied as potential markers of early Abiraterone response in mCRPC patients.

## 1. Introduction

During the past few years, there has been significant progress in treatment options for metastatic castration resistant prostate cancer (mCRPC). After prostate cancer develops, resistance to androgen-deprivation therapy (ADT), patients can be treated with different agents including chemotherapy, next generation ADT drugs, Sipuleucel-T or Radium-223 [1,2,3,4,5,6,7,8]. Amongst these, Abiraterone acetate (Abiraterone), a selective CYP450 17A1 inhibitor, is available in the pre- and post-chemotherapy setting and is broadly accepted as a standard of care due to its life-prolonging potential and generally low toxicity [1,2,9].

A challenging aspect for clinicians in this setting is the early determination of treatment success, particularly the early identification of response or failure in patients whose lack of symptoms makes a clinical decision difficult. Except a decline in total prostate specific antigen (tPSA), there are no easily available prognostic biomarkers besides clinical parameters to determine therapeutic success. Therefore, the decision whether to continue or to change a therapy regime is mainly based on clinical factors (e.g., eastern cooperative oncology group performance status (ECOG)) and tPSA, which is still the most commonly used therapy control marker in mCRPC.

However, there are several limitations concerning tPSA, especially in mCRPC. The parameter does not always decline right away even though patients might still benefit from therapy. Thus, in the registration trials, tPSA could not be validated as an independent prognosticator for therapy response [1,2]. Furthermore, an early rise of tPSA during the first 12 weeks of therapy followed by a delayed decline (PSA-flare) during Abiraterone-therapy was recently found to be a prognosticator for an improved median progression free survival (PFS) [10]. PSA-flare occurs in about 10% of the patients treated with Abiraterone. A larger proportion of patients with initial increase of tPSA (nearly 50%), however, will suffer a continuous rise of tPSA over time followed by clinical progression [10]. In clinical routines, the differentiation between PSA-flare and continuous rise is difficult, resulting in either early, sometimes premature, interruption of therapy or waiting for the potential benefit of a delayed tPSA-decline with, in many cases, a treatment beyond true progression. Therefore, additional biomarkers to distinguish between true progression and a delayed tPSA response are desperately needed for these situations.

Several biomarkers, including circulating tumor cells (CTCs) and lactate dehydrogenase (LDH), have recently been discussed as response-indicators [11,12].

CTC counts at baseline are considered to be prognostic for the duration of treatment with a resulting shorter time of treatment for ≥5 CTCs [11]. However, the sensitivity of detection of CTCs is limited in patients with low tumor load and differs depending on the site of metastasis [13]. Additionally, CTC-assays are expensive and not broadly available.

Similar data show that the combination of ≥5 CTC count and an elevated LDH level after 12 weeks of therapy may be a surrogate for poor overall survival (OS) [12]. However, for broad acceptance and use, more easily available as well as more sensitive, validated and non-expensive assays are required [14].

In 1991, the low specificity of PSA for the detection of prostate cancer was improved by the identification of two major molecular subforms of PSA, an unbound proportion (free PSA, fPSA) and a form complexed with the protease inhibitor alpha-1-antichimotrypsin [15]. It was shown that the chance of detecting prostate cancer was lower with increasing proportion of fPSA (%fPSA) [16,17]. Following the detection of precursor forms of fPSA (proPSA), further investigation concluded that proPSA was associated with prostate cancer with a higher probability of cancer as the percentage of proPSA in fPSA increases [18,19]. Within the proPSA fraction, further discrimination could be made by the detection of truncated forms which are more resistant to activation to mature PSA, with [−2]proPSA being the most consistent of these [20]. According to recent investigations, the ratio of [−2]proPSA to fPSA (%[−2]proPSA) and the Prostate Health Index (PHI = ([−2]proPSA/fPSA) × √tPSA) seem to be the strongest predictors of prostate cancer compared to tPSA and its subforms [21,22]. In addition, there was evidence for a correlation with more aggressive variants of prostate cancer since the variables increased with increasing Gleason-Score [23,24].

In this study, we aimed to investigate whether PSA subforms could be helpful for predicting treatment response and prognosticating survival outcomes in patients with mCRPC treated with Abiraterone. When added to tPSA in the setting of organ confined prostate cancer, the Supplementary Materials of fPSA, [−2]proPSA and PHI help to diagnose and identify more aggressive forms of prostate cancer [25,26]. Therefore, we assumed that these markers could also be useful to facilitate decision making in the treatment of mCRPC.

## 2. Results

### 2.1. Characteristics of the Study Group

For the patients alive at the time of analysis, the median follow-up was 25 months (Interquartile range (IQR) 14.5–28.0) in July 2015. The median time on Abiraterone-therapy was 13 months (IQR 10.5–19.0) with six (24%) patients on therapy at the time of last data acquisition. No dose modifications were necessary for any patients. Descriptive characteristics of the cohort are given in Table 1. The median age of the patients was 71 years (IQR 63–74). Bone metastases were present in 76%, lymphonodal metastases in 64% and visceral metastases in 8% of patients at the beginning of Abiraterone-therapy. An unfavorable Gleason-Score of ≥8 at initial diagnosis of PCa was found in 60%. Median baseline levels were 61.7 ng/mL for tPSA (IQR 29.0–299.5), 11.2 ng/mL for fPSA (IQR 5.2–29.1), 485.3 pg/mL for [−2]proPSA (IQR 272.8–1107.9) and 327.3 for PHI (IQR 212.5–612.0). No patient showed elevated liver enzyme- or creatinine-concentrations during the course of the study.

Antiresorptive therapy (Zoledronic acid or Denosumab) was administered in 12 patients (48%). All of these remained on a stable dose of the drug during the course of Abiraterone-therapy.

### 2.2. Value of PSA Subforms as Prognostic Markers

**Figure 1:**
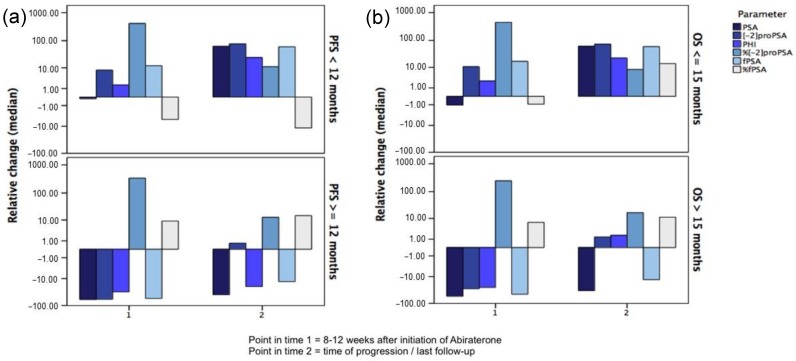
Relative median changes of PSA subforms at 8–12 weeks and at progression or last follow-up compared to baseline for (**a**) PFS < 12 months vs. ≥12 months; and (**b**) OS ≤ 15 months vs. >15 months.

Alterations of the baseline values are presented as relative change of median values in percent (Figure 1). The definition of progressive disease is explained in paragraph 4 (Materials and Methods). For patients with shorter response to therapy (PFS < 12 months and OS ≤ 15 months) the median fPSA and [−2]proPSA increased at 8–12 weeks. In contrast to these two subforms, the median tPSA showed stable values at the same time for these patients. The other parameters (PHI, %[−2]proPSA and %fPSA) seem to play a minor role, considering the scope of our study, since changes did not differ between the groups (Table 2).

In patients with < vs. ≥12 months of PFS, the relative change of median tPSA values (−0.1% vs. −86.8%; *p* = 0.02 (Mann–Whitney–Wilcoxon Test)) at 8–12 weeks compared to baseline differed significantly. Similar results were shown for decreasing [−2]proPSA- (8.1% vs. −59.3%; *p* = 0.05) and fPSA values (12.1% vs. −55.3%; *p* = 0.03), respectively.

For men with ≤ vs. >15 months of OS, there was a non-significant trend for a difference in relative changes of median fPSA values (17.0% vs. −46.3%; *p* = 0.06). Considering OS, no conclusive change of median values was seen for the other parameters (tPSA, [−2]proPSA, PHI, %[−2]proPSA and %fPSA).

In univariate Cox-regression analysis, rising [−2]proPSA and fPSA were non-significant prognosticators of shorter PFS (Hazard ratio (HR): 2.0, (95% confidence interval (CI): 0.8–4.8); *p* = 0.14 for both parameters). Analogously, the results for OS showed a non-significant trend for rising [−2]proPSA and fPSA regarding the prognostication of poor OS (HR: 2.5 (95% CI: 0.7–9.1); *p* = 0.16 for both parameters). In this analysis, PHI also showed a trend towards shortened PFS in case of an early increase (HR: 2.0 (95% CI: 0.9–5.0); *p* = 0.11). For OS, however, PHI did not show any trend for shortened survival (HR: 1.5 (95% CI: 0.4–5.4); *p* = 0.49). Of note, rising tPSA changes did not prove to be a prognosticator for worse PFS (HR: 1.6 (95% CI: 0.6–4.0); *p* = 0.29) or OS (HR: 1.3 (95% CI: 0.4–4.5); *p* = 0.72) (Table 3).

### 2.3. Kaplan–MEIER Survival Analysis

**Figure 2:**
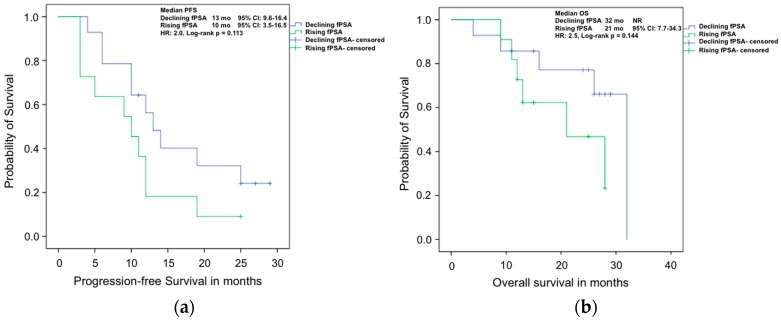
Kaplan–Meier analyses of (**a**) progression free survival; and (**b**) overall survival of mCRPC patients treated with Abiraterone with rising or declining fPSA values after 8–12 weeks of therapy.

**Figure 3:**
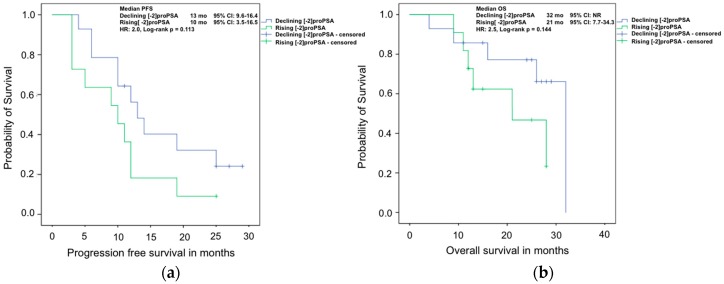
Kaplan–Meier analyses of (**a**) progression free survival; and (**b**) overall survival of mCRPC patients treated with Abiraterone with rising or declining [−2]proPSA values after 8–12 weeks of therapy.

**Figure 4:**
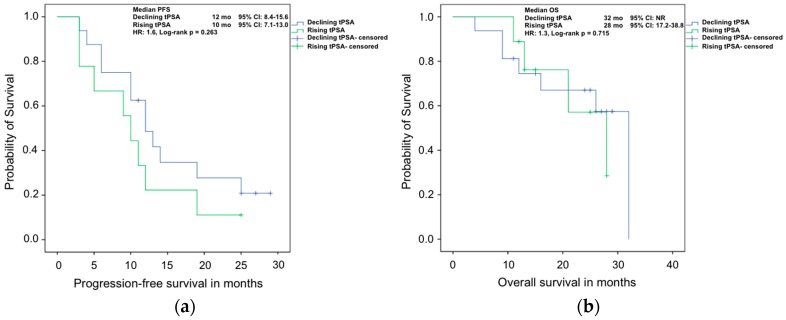
Kaplan–Meier analyses of (**a**) progression free survival; and (**b**) overall survival of mCRPC patients treated with Abiraterone with rising or declining tPSA values after 8–12 weeks of therapy.

The Kaplan–Meier analyses for PFS and OS are presented in Figure 2, Figure 3 and Figure 4. Here, the strongest marker for longer PFS in Mann–Whitney–Wilcoxon comparison of median values, declining fPSA, also showed better survival outcomes. A declining fPSA value at 8–12 weeks was associated with a median PFS of 13 months (95% CI: 9.6–16.4 months) compared to 10 months (95% CI: 3.5–16.5 months) in patients with increasing values (log-rank *p* = 0.11).

Correspondingly, in the analysis of OS, a decreasing fPSA at 8–12 weeks of therapy indicated a median OS of 32 months (95% CI: not reached (NR)) compared to 21 months (95% CI: 7.7–34.3 months) in men with rising fPSA (*p* = 0.14) (Figure 2).

The Kaplan–Meier analysis of PFS and OS with declining and rising [−2]proPSA showed similar results (Figure 3).

In contrast, in the analysis of PFS for tPSA, there was less difference between rising and declining values. A tPSA decrease was associated with a median PFS of 12 months (95% CI: 8.4–15.6 months) compared to 10 months (95% CI: 7.1–13.0 months) in the case of increasing values (*p* = 0.26). Correspondingly, the evaluation of OS did not show differences between tPSA values in both groups with 32 months (95% CI: NR) vs. 28 months (95% CI: 17.2–38.8 months, *p* = 0.72) (Figure 4).

## 3. Discussion

To our knowledge, the relevance of fPSA and [−2]proPSA as well as PHI in patients with mCRPC under Abiraterone has not been investigated thus far. Standard monitoring of therapy is mainly based on the measurement of tPSA as well as radiological criteria and the general condition of the patients like the assessment of ECOG performance status [27].

Among the group of patients with mCRPC, a large proportion of up to 90% shows bone metastases [28]. During early imaging, especially in the first three to six months of therapy, an early “bone-flare“ can occur in a relevant amount of patients, which can lead to incorrect classification as progressive disease (PD) [29,30].

The most common parameter being used for determination of treatment success under any therapy for mCRPC is tPSA. Here, comparable to the above-mentioned “bone-flare”, a “PSA-flare” can occur under therapy with Abiraterone [10]. These common phenomena of everyday practice leave the burden to decide whether a therapy should be terminated or continued to the clinician. This dilemma holds especially true in asymptomatic or oligosymptomatic patients, since lack of clinical information can render decision making in some cases impossible. Therefore, in the case of biochemical progression only, current guidelines recommend continuing treatment until unequivocal information on progression is evident [31].

The data concerning monitoring of response or progression at an early stage of treatment are contradictory. PSA-flare has been reported to occur under other therapies than Abiraterone, like Docetaxel [32] and could be caused by a delayed response or rapid cell destruction with release of tPSA to the circulation [10]. Under treatment with Abiraterone, immediate or delayed tPSA-responses are common, but there are patients who will not show a tPSA-decline at all [10,33]. In our cohort, as well, a PSA-flare was seen in three patients, which is in line with the previously described 10% in other cohorts [10]. These three patients showed various duration of response to Abiraterone and stayed under treatment for nine, 26 and 38 months. Thus, the early differentiation between “PSA-flare” and true PD is difficult and remains mostly unresolved. This clinical dilemma can be even more complex if a possible “bone-flare” is suspected [29,30].

Although a significant tPSA decline of at least 50% from baseline was found in a large proportion of the patients treated within the registration trials for Abiraterone (COU-AA-301 and COU-AA-302), tPSA-changes were not found to be independent prognosticators for survival outcomes [1,2]. These non-straightforward findings are in line with our results. In our cohort, the median change of tPSA at 8–12 weeks of Abiraterone-therapy was a prognosticator for a PFS < 12 vs. ≥12 months (*p* = 0.02) with a decline resulting in a longer PFS, but the univariate cox-regression analysis showed no significant association (HR 1.6, *p* = 0.29); this may be due to our relatively small cohort. For this reason, no multivariate analysis was performed.

Some alternative biomarkers were recently evaluated. In contrast to tPSA, CTCs do not show a “flare” phenomenon and therefore could be ideal as a surrogate for OS [34]. In this context, several studies reported that the post-therapy CTC enumeration could be considered to be an independent prognostic parameter for OS [9,35]. However, due to costs, a relevant rate of falsely negative results and a lack of easily available assays the broad use of CTC enumeration remains limited [34].

Additionally, few retrospective studies report a trend for baseline LDH values above the range of normal limits being inversely associated with survival outcomes [12,35]. However, LDH is a very unspecific biomarker of cell necrosis in many tumor types including mCRPC and probably only reflects tumor burden as well as cell turnover as a marker of cancer aggressiveness [34].

Because of this lack of broadly available surrogate markers, we analyzed the potential of readily accessible biomarkers that are already being used in different settings within prostate cancer.

Until now, the role of fPSA is limited to the diagnosis of prostate cancer [19]. A lower proportion of %fPSA can be found in men with prostate cancer compared to patients with benign prostate enlargement [36]. Clinicians aim to avoid unnecessary biopsies by the measurement of fPSA, especially in cases with a mild tPSA elevation of 4–10 ng/mL. Additionally, a correlation between fPSA and the aggressiveness of the prostate cancers was shown [37].

More recent investigations demonstrated that this correlation with aggressiveness is even higher for the subform-proportion of %[−2]proPSA and PHI [23,24]. In addition, both parameters are currently considered to be the strongest predictors for presence of prostate cancer [21,22,23,38] with a higher specificity compared to fPSA and tPSA [22].

Due to the correlation between PSA subforms and the aggressiveness of the tumor we assumed that these parameters could play a role as surrogate biomarkers under therapy of mCRPC as well. Our results underline the non-straightforward data regarding early rising tPSA measurements with no validity concerning therapy outcomes. In contrast, we were able to show a trend towards rising [−2]proPSA, fPSA and, less importantly, PHI in patients with shorter OS and PFS at an early stage of treatment. Compared to tPSA, we need to consider that tPSA was taken into account for the definition of PD during treatment with Abiraterone. This might be an explanation for the less obvious separation of the curves during Kaplan–Meier analyses when comparing tPSA to fPSA and [−2]proPSA. However, our data suggest that fPSA and [−2]proPSA might be further studied as potential parameters for the prognostication of therapy response under early treatment with Abiraterone in case of non-straightforward development of PSA-values. The relative changes of both, median [−2]proPSA and fPSA values, were shown to be almost equal.

Our study is limited due to its relatively small cohort of only 25 patients recruited in a single center. Further larger studies are needed to explore the potential prognostic and predictive impact of fPSA and [−2]proPSA in mCRPC treated with Abiraterone.

## 4. Materials and Methods

### 4.1. Patients

Twenty-five patients with mCRPC presenting at the Prostate Center of the University Hospital Muenster (Münster, Germany), and receiving Abiraterone-therapy between March 2012 and July 2014, were reviewed. They had given written informed consent before participating and ethics committee-approval was granted for this study.

All patients presented with confirmed mCRPC, defined by prostate cancer working group 2 (PCWG2) criteria [31], in either pre- or post-chemotherapy setting (20 of them received Abiraterone in a pre- and 5 in a post-chemotherapy setting) suitable for Abiraterone-treatment. The pre-chemotherapy patients were asymptomatic or oligo-symptomatic, not requiring opiates and had a pain level of no more than 3 out of 10 on the numeric-rating-scale. Patients receiving Abiraterone in the post-chemotherapy setting had previously documented PD either on or after docetaxel chemotherapy. One of the 25 patients had received Enzalutamide previously. All patients in our study were either on a stable dose of an antiresorptive agent (i.e., Zoledronic acid or Denosumab) at least three months prior to the start of Abiraterone and during the whole treatment phase or did not receive antiresorptive medication at all. The patients were not systematically followed up with 12-weekly imaging when no PD was suspected, but when biochemical progression was evident, or when clinical progression occurred, even when there was no biochemical progression. CT-, MRI- or PSMA-PET-CT-scans of thorax, abdomen and pelvis were used for the evaluation of soft tissue metastases. Bone scans were done to acquire additional information on baseline bone metastases. PD was defined by RECIST 1.1 criteria [39] for cross-sectional imaging and by PCWG2 criteria for bone scans [31].

A physician with large expertise in the treatment of mCRPC assessed the current response status i.e., complete remission (CR), partial remission (PR), stable disease (SD) or PD at each visit. For defining PD, deterioration of general condition, worsening of pain or laboratory constellations (rising tPSA) and imaging were taken into account, according to PCWG2 criteria. Rising tPSA alone was not sufficient to define progression. The results of PSA-subform analysis, which was done in a retrospective setting, were not taken into consideration for therapeutic decisions.

All patients presented the day before the start of Abiraterone-therapy to have blood drawn for baseline analysis, two and four weeks after initiation of therapy and every four weeks thereafter.

### 4.2. Analysis of PSA Subforms

Blood samples were allowed to clot at room temperature and then centrifuged at 1600× *g* for 15 min at 4 °C. Taking into account the stability issue of [−2]proPSA, serum samples were frozen at −80 °C within 3 h after blood draw [40]. After thawing of the frozen samples at room temperature, samples were processed immediately and used to determine tPSA, fPSA, [−2]proPSA on the Access II instrument, WHO-calibrated (Beckman Coulter, Krefeld, Germany) for the timepoints ‘baseline’, ‘8–12 weeks under therapy’ and ‘progression’. Furthermore, %fPSA, %[−2]proPSA and PHI were calculated.

We carried out the analyses according to the manufacturers’ instructions on the AccessII-instrument (Beckman-Coulter, Krefeld, Germany)—batch numbers: tPSA (470206), fPSA (470202) and [−2]proPSA (434426). The results for PSA-subforms exceeded the measuring range of the instrument in many cases, with [−2]proPSA values up to 8000 pg/mL. Therefore, dilution was necessary. Due to a nonlinear response with serial dilution of the proPSA kit diluent, we used the LowCross-Buffer^®^ (CANDOR Bioscience GmbH, Wangen, Germany, lot number 100A766f) as sample diluent in order to reduce nonspecific binding and cross-reactivities of the antibodies and matrix effects from blood serum. This procedure helped to minimize interferences in this assay and improves the reliability of the results. When dilution of serum samples was necessary due to very high [−2]proPSA values, all the other parameters were measured within the same diluted sample.

### 4.3. Statistical Methods

The descriptive statistics are reported as medians with IQR for continuous variables and as populations and frequencies for categorical variables. We performed the Mann–Whitney–Wilcoxon Test to determine the significance of the differences between categorical and continuous variables, respectively. We used the Kaplan–Meier-estimates for survival analyses. Univariate analysis of the different biomarkers was done with Cox-regression-models. Hazard ratios are given with 95% confidence intervals. All reported *p*-values are two-sided and statistical significance was assumed with a *p* ≤ 0.05. SPSS-Statistics V.22 (IBM Inc., Armonk, NY, USA) was used for statistical assessment.

## 5. Conclusions

When added to tPSA information, which is traditionally used as therapy response indicator, changes of fPSA and [−2]proPSA at 8–12 weeks of Abiraterone-therapy may be a promising therapy control marker to help physicians make meaningful decisions on whether to stop or to continue Abiraterone-therapy in men with mCRPC but need to be further studied. Due to its better availability, the measurement of fPSA seems to be more feasible than [−2]proPSA.

## Figures and Tables

**Table 1:** Baseline characteristics of patients with mCRPC under therapy with Abiraterone IQR, interquartile range; ECOG, eastern cooperative oncology group performance status.

Variable	Number
Patients (*n*) (%)	25 (100)
Age, median (years) (IQR)	71.0 (62.5–74.0)
Median follow-up (months) (IQR)	25.0 (14.5–28.0)
Median duration of therapy (months) (IQR)	13.0 (10.5–19.0)
Presence of lymphnode metastases (*n*) (%)	16 (64.0)
Presence of bone metastases (*n*) (%)	19 (76.0)
Presence of visceral metastases (*n*) (%)	2 (8.0)
Pre chemotherapy (*n*) (%)	20 (80.0)
Post chemotherapy (*n*) (%)	5 (20.0)
Antiresorptive therapy (*n*) (%)	12 (48.0)
Zoledronic acid (*n*) (%)	7 (28.0)
Denosumab (*n*) (%)	5 (20.0)
ECOG (all) (*n*) (%)	
0–1	22 (88.0)
>1	3 (12.0)
Gleason Score ≥ 8 (*n*) (%)	15 (60.0)
Median tPSA baseline (ng/mL) (IQR)	61.7 (29.0–299.5)
Median fPSA baseline (ng/mL) (IQR)	11.2 (5.2–29.1)
Median [−2]proPSA baseline (pg/mL) (IQR)	485.3 (272.8–1107.9)
Median PHI baseline (IQR)	327.3 (212.5–612.0)
Best clinical outcome (*n*) (%)	
Complete remission	1 (4.0)
Partial remission	10 (40.0)
Stable disease	10 (40.0)
Progressive disease	4 (16.0)
tPSA decrease ≥ 50% (*n*) (%)	12 (48.0)
tPSA decrease ≥ 90% (*n*) (%)	7 (28.0)
Patients died (*n*) (%)	11 (44.0)

**Table 2:** Relative changes of median PSA-subform values (%) in 25 patients at 8–12 weeks for OS and PFS OS, overall survival; PFS, progression free survival.

**Variable**	**OS ≤ 15 Months**	**OS > 15 Months**	***p***
tPSA	−1.1 (−54.5–66.3)	−55.1 (−74.6–13.2)	0.20
[−2]proPSA	10.5 (−50.7–62.0)	−29.1 (−66.9–19.9)	0.15
PHI	2.6 (−42.9–39.0)	−25.9 (−41.4–19.0)	0.32
%[−2]proPSA	449.0 (260.6–588.4)	242.1 (149.5–577.7)	0.27
fPSA	17.0 (−41.8–50.3)	−46.3 (−72.0–8.1)	0.06
%fPSA	−0.9 (−19.6–28.1)	7.0 (−27.6–41.3)	0.61
	**PFS < 12 Months**	**PFS ≥ 12 Months**	
tPSA	−0.1 (−0.1–71.3)	−86.8 (−60.9–9.4)	0.02
[−2]proPSA	8.1 (−21.2–60.1)	−59.3 (−74.0–18.5)	0.05
PHI	1.7 (−24.4–63.6)	−32.1 (−47.8–4.1)	0.12
%[−2]proPSA	416.0 (159.8–754.2)	335.5 (163.2–550.7)	0.73
fPSA	12.1 (−22.7–43.8)	−55.3 (−83.3–13.6)	0.03
%fPSA	−5.2 (−28.3–16.7)	9.1 (−17.3–42.0)	0.15

**Table 3:** Changes of median PSA-subform values in univariate analysis OS, overall survival; PFS, progression; HR, hazard ratio; CI, confidence interval.

Univariate Analysis for OS			Univariate Analysis for PFS		
Variable	HR (95% CI)	*p*	Variable	HR (95% CI)	*p*
tPSA increase	1.3 (0.4–4.5)	0.72	tPSA increase	1.6 (0.6–4.0)	0.29
yes vs. no			yes vs. no		
fPSA increase	2.5 (0.7–9.1)	0.16	fPSA increase	2.0 (0.8–4.8)	0.14
yes vs. no			yes vs. no		
[−2]proPSA increase	2.5 (0.7–9.1)	0.16	[−2]proPSA increase	2.0 (0.8–4.8)	0.14
yes vs. no			yes vs. no		
PHI increase	1.5 (0.4–5.4)	0.49	PHI increase	2.0 (0.9–5.0)	0.11
yes vs. no			yes vs. no		
%[−2]proPSA increase	23.1 (0–∞)	0.57	%[−2]proPSA increase	1.8 (0.2–13.5)	0.57
yes vs. no			yes vs. no		
%fPSA increase	0.4 (0.1–1.7)	0.23	%fPSA increase	1.2 (0.5–2.8)	0.73
no vs. yes			no vs. yes		

## Supplementary Materials

Supplementary materials can be found at www.mdpi.com/1422-0067/17/9/1520/s1.

## Abbreviations

mCRPC	metastatic Castration Resistant Prostate Cancer
ADT	Androgen-Deprivation Therapy
PSA	Prostate Specific Antigen
tPSA	total PSA
ECOG	Eastern Cooperative Oncology Group performance status
PFS	Progression Free Survival
CTC	Circulating Tumor Cells
LDH	Lactate Dehydrogenase
OS	Overall Survival
fPSA	free PSA
PHI	Prostate Health Index
IQR	Interquartile Range
HR	Hazard Ratio
CI	Confidence Interval
NR	Not Reached
PD	Progressive Disease
PCWG2	Prostate Cancer Working Group 2
CR	Complete Remission
PR	Partial Remission
SD	Stable Disease

## References

[1] Ryan C.J., Smith M.R., Fizazi K., Saad F., Mulders P.F., Sternberg C.N., Miller K., Logothetis C.J., Shore N.D., Small E.J. (2015). Abiraterone acetate plus prednisone versus placebo plus prednisone in chemotherapy-naive men with metastatic castration-resistant prostate cancer (COU-AA-302): Final overall survival analysis of a randomised, double-blind, placebo-controlled phase 3 study. Lancet Oncol..

[2] Logothetis C.J., Basch E., Molina A., Fizazi K., North S.A., Chi K.N., Jones R.J., Goodman O.B., Mainwaring P.N., Sternberg C.N. (2012). Effect of abiraterone acetate and prednisone compared with placebo and prednisone on pain control and skeletal-related events in patients with metastatic castration-resistant prostate cancer: Exploratory analysis of data from the COU-AA-301 randomised trial. Lancet Oncol..

[3] Kantoff P.W., Higano C.S., Shore N.D., Berger E.R., Small E.J., Penson D.F., Redfern C.H., Ferrari A.C., Dreicer R., Sims R.B. (2010). Sipuleucel-t immunotherapy for castration-resistant prostate cancer. N. Engl. J. Med..

[4] Loriot Y., Miller K., Sternberg C.N., Fizazi K., de Bono J.S., Chowdhury S., Higano C.S., Noonberg S., Holmstrom S., Mansbach H. (2015). Effect of enzalutamide on health-related quality of life, pain, and skeletal-related events in asymptomatic and minimally symptomatic, chemotherapy-naive patients with metastatic castration-resistant prostate cancer (prevail): Results from a randomised, phase 3 trial. Lancet Oncol..

[5] Fizazi K., Scher H.I., Miller K., Basch E., Sternberg C.N., Cella D., Forer D., Hirmand M., de Bono J.S. (2014). Effect of enzalutamide on time to first skeletal-related event, pain, and quality of life in men with castration-resistant prostate cancer: Results from the randomised, phase 3 affirm trial. Lancet Oncol..

[6] Berthold D.R., Pond G.R., Soban F., de Wit R., Eisenberger M., Tannock I.F. (2008). Docetaxel plus prednisone or mitoxantrone plus prednisone for advanced prostate cancer: Updated survival in the TAX 327 study. J. Clin. Oncol..

[7] Petrylak D.P., Tangen C.M., Hussain M.H., Lara P.N., Jones J.A., Taplin M.E., Burch P.A., Berry D., Moinpour C., Kohli M. (2004). Docetaxel and estramustine compared with mitoxantrone and prednisone for advanced refractory prostate cancer. N. Engl. J. Med..

[8] Sartor O., Coleman R., Nilsson S., Heinrich D., Helle S.I., O’Sullivan J.M., Fossa S.D., Chodacki A., Wiechno P., Logue J. (2014). Effect of radium-223 dichloride on symptomatic skeletal events in patients with castration-resistant prostate cancer and bone metastases: Results from a phase 3, double-blind, randomised trial. Lancet Oncol..

[9] De Bono J.S., Logothetis C.J., Molina A., Fizazi K., North S., Chu L., Chi K.N., Jones R.J., Goodman O.B., Saad F. (2011). Abiraterone and increased survival in metastatic prostate cancer. N. Engl. J. Med..

[10] Burgio S.L., Conteduca V., Rudnas B., Carrozza F., Campadelli E., Bianchi E., Fabbri P., Montanari M., Carretta E., Menna C. (2015). PSA flare with abiraterone in patients with metastatic castration-resistant prostate cancer. Clin. Genitourin. Cancer.

[11] Danila D.C., Morris M.J., de Bono J.S., Ryan C.J., Denmeade S.R., Smith M.R., Taplin M.E., Bubley G.J., Kheoh T., Haqq C. (2010). Phase II multicenter study of abiraterone acetate plus prednisone therapy in patients with docetaxel-treated castration-resistant prostate cancer. J. Clin. Oncol..

[12] Scher H.I., Heller G., Molina A., Attard G., Danila D.C., Jia X., Peng W., Sandhu S.K., Olmos D., Riisnaes R. (2015). Circulating tumor cell biomarker panel as an individual-level surrogate for survival in metastatic castration-resistant prostate cancer. J. Clin. Oncol..

[13] Danila D.C., Heller G., Gignac G.A., Gonzalez-Espinoza R., Anand A., Tanaka E., Lilja H., Schwartz L., Larson S., Fleisher M. (2007). Circulating tumor cell number and prognosis in progressive castration-resistant prostate cancer. Clin. Cancer Res..

[14] Danila D.C., Fleisher M., Scher H.I. (2011). Circulating tumor cells as biomarkers in prostate cancer. Clin. Cancer Res..

[15] Lilja H., Christensson A., Dahlen U., Matikainen M.T., Nilsson O., Pettersson K., Lovgren T. (1991). Prostate-specific antigen in serum occurs predominantly in complex with α 1-antichymotrypsin. Clin. Chem..

[16] Lilja H., Stenman U.H. (1996). Successful separation between benign prostatic hyperplasia and prostate cancer by measurement of free and complexed psa. Cancer Treat. Res..

[17] Stenman U.H., Leinonen J., Alfthan H., Rannikko S., Tuhkanen K., Alfthan O. (1991). A complex between prostate-specific antigen and alpha 1-antichymotrypsin is the major form of prostate-specific antigen in serum of patients with prostatic cancer: Assay of the complex improves clinical sensitivity for cancer. Cancer Res..

[18] Mikolajczyk S.D., Rittenhouse H.G. (2003). Pro psa: A more cancer specific form of prostate specific antigen for the early detection of prostate cancer. Keio J. Med..

[19] Tosoian J., Loeb S. (2010). Psa and beyond: The past, present, and future of investigative biomarkers for prostate cancer. Sci. World J..

[20] Mikolajczyk S.D., Marks L.S., Partin A.W., Rittenhouse H.G. (2002). Free prostate-specific antigen in serum is becoming more complex. Urology.

[21] Lazzeri M., Briganti A., Scattoni V., Lughezzani G., Larcher A., Gadda G.M., Lista G., Cestari A., Buffi N., Bini V. (2012). Serum index test %[−2]proPSA and prostate health index are more accurate than prostate specific antigen and %fPSA in predicting a positive repeat prostate biopsy. J. Urol..

[22] Catalona W.J., Partin A.W., Sanda M.G., Wei J.T., Klee G.G., Bangma C.H., Slawin K.M., Marks L.S., Loeb S., Broyles D.L. (2011). A multicenter study of [−2]pro-prostate specific antigen combined with prostate specific antigen and free prostate specific antigen for prostate cancer detection in the 2.0 to 10.0 ng/mL prostate specific antigen range. J. Urol..

[23] Lazzeri M., Haese A., Abrate A., de la Taille A., Redorta J.P., McNicholas T., Lughezzani G., Lista G., Larcher A., Bini V. (2013). Clinical performance of serum prostate-specific antigen isoform [−2]proPSA (p2PSA) and its derivatives, %p2PSA and the prostate health index (PHI), in men with a family history of prostate cancer: Results from a multicentre european study, the prometheus project. BJU Int..

[24] Stephan C., Jung K., Semjonow A., Schulze-Forster K., Cammann H., Hu X., Meyer H.A., Bogemann M., Miller K., Friedersdorff F. (2013). Comparative assessment of urinary prostate cancer antigen 3 and TMPRSS2: Erg gene fusion with the serum [−2]proprostate-specific antigen-based prostate health index for detection of prostate cancer. Clin. Chem..

[25] Fossati N., Buffi N.M., Haese A., Stephan C., Larcher A., McNicholas T., de la Taille A., Freschi M., Lughezzani G., Abrate A. (2015). Preoperative prostate-specific antigen isoform p2PSA and its derivatives, %p2PSA and prostate health index, predict pathologic outcomes in patients undergoing radical prostatectomy for prostate cancer: Results from a multicentric european prospective study. Eur. Urol..

[26] Fossati N., Lazzeri M., Haese A., McNicholas T., de la Taille A., Buffi N.M., Lughezzani G., Gadda G.M., Lista G., Larcher A. (2015). Clinical performance of serum isoform [−2]proPSA (p2PSA), and its derivatives %p2PSA and the prostate health index, in men aged <60 years: Results from a multicentric european study. BJU Int..

[27] Woo H.H., Begbie S., Gogna K., Mainwaring P.N., Murphy D.G., Parnis F., Steer C., Davis I.D. (2014). Multidisciplinary consensus: A practical guide for the integration of abiraterone into clinical practice. Asia Pac. J. Clin. Oncol..

[28] Roghmann F., Antczak C., McKay R.R., Choueiri T., Hu J.C., Kibel A.S., Kim S.P., Kowalczyk K.J., Menon M., Nguyen P.L. (2015). The burden of skeletal-related events in patients with prostate cancer and bone metastasis. Urol. Oncol..

[29] Ryan C.J., Shah S., Efstathiou E., Smith M.R., Taplin M.E., Bubley G.J., Logothetis C.J., Kheoh T., Kilian C., Haqq C.M. (2011). Phase II study of abiraterone acetate in chemotherapy-naive metastatic castration-resistant prostate cancer displaying bone flare discordant with serologic response. Clin. Cancer Res..

[30] Johns W.D., Garnick M.B., Kaplan W.D. (1990). Leuprolide therapy for prostate cancer: An association with scintigraphic “flare” on bone scan. Clin. Nucl. Med..

[31] Scher H.I., Halabi S., Tannock I., Morris M., Sternberg C.N., Carducci M.A., Eisenberger M.A., Higano C., Bubley G.J., Dreicer R. (2008). Design and end points of clinical trials for patients with progressive prostate cancer and castrate levels of testosterone: Recommendations of the prostate cancer clinical trials working group. J. Clin. Oncol..

[32] Berthold D.R., Pond G.R., Roessner M., de Wit R., Eisenberger M., Tannock A.I. (2008). TAX-327 investigators Treatment of hormone-refractory prostate cancer with docetaxel or mitoxantrone: Relationships between prostate-specific antigen, pain, and quality of life response and survival in the TAX-327 study. Clin. Cancer Res..

[33] Leibowitz-Amit R., Templeton A.J., Omlin A., Pezaro C., Atenafu E.G., Keizman D., Vera-Badillo F., Seah J.A., Attard G., Knox J.J. (2014). Clinical variables associated with psa response to abiraterone acetate in patients with metastatic castration-resistant prostate cancer. Ann. Oncol..

[34] Armstrong A.J., Eisenberger M.A., Halabi S., Oudard S., Nanus D.M., Petrylak D.P., Sartor A.O., Scher H.I. (2012). Biomarkers in the management and treatment of men with metastatic castration-resistant prostate cancer. Eur. Urol..

[35] Scher H.I., Jia X., de Bono J.S., Fleisher M., Pienta K.J., Raghavan D., Heller G. (2009). Circulating tumour cells as prognostic markers in progressive, castration-resistant prostate cancer: A reanalysis of IMMC38 trial data. Lancet Oncol..

[36] De Angelis G., Rittenhouse H.G., Mikolajczyk S.D., Blair Shamel L., Semjonow A. (2007). Twenty years of PSA: From prostate antigen to tumor marker. Rev. Urol..

[37] Catalona W.J., Partin A.W., Slawin K.M., Brawer M.K., Flanigan R.C., Patel A., Richie J.P., deKernion J.B., Walsh P.C., Scardino P.T. (1998). Use of the percentage of free prostate-specific antigen to enhance differentiation of prostate cancer from benign prostatic disease: A prospective multicenter clinical trial. JAMA.

[38] Abrate A., Lughezzani G., Gadda G.M., Lista G., Kinzikeeva E., Fossati N., Larcher A., Dell’Oglio P., Mistretta F., Buffi N. (2014). Clinical use of [−2]proPSA (p2PSA) and its derivatives (%p2PSA and prostate health index) for the detection of prostate cancer: A review of the literature. Korean J. Urol..

[39] Eisenhauer E.A., Therasse P., Bogaerts J., Schwartz L.H., Sargent D., Ford R., Dancey J., Arbuck S., Gwyther S., Mooney M. (2009). New response evaluation criteria in solid tumours: Revised recist guideline (version 1.1). Eur. J. Cancer.

[40] Semjonow A., Kopke T., Eltze E., Pepping-Schefers B., Burgel H., Darte C. (2010). Pre-analytical in vitro stability of [−2]proPSA in blood and serum. Clin. Biochem..

